# A Novel Semi-Supervised Methodology for Extracting Tumor Type-Specific MRS Sources in Human Brain Data

**DOI:** 10.1371/journal.pone.0083773

**Published:** 2013-12-23

**Authors:** Sandra Ortega-Martorell, Héctor Ruiz, Alfredo Vellido, Iván Olier, Enrique Romero, Margarida Julià-Sapé, José D. Martín, Ian H. Jarman, Carles Arús, Paulo J. G. Lisboa

**Affiliations:** 1 Department of Mathematics and Statistics, Liverpool John Moores University, Liverpool, United Kingdom; 2 Department of Computer Languages and Systems, Universitat Politècnica de Catalunya - BarcelonaTech, Barcelona, Spain; 3 Institute of Population Health, The University of Manchester, Manchester, United Kingdom; 4 Centro de Investigación Biomédica en Red en Bioingeniería, Biomateriales y Nanomedicina (CIBER-BBN), Cerdanyola del Vallès, Spain; 5 Departament de Bioquímica i Biología Molecular, Universitat Autònoma de Barcelona, Cerdanyola del Vallès, Spain; 6 Institut de Biotecnologia i de Biomedicina, Universitat Autònoma de Barcelona, Cerdanyola del Vallès, Spain; 7 Departamento de Ingeniería Electrónica, Universidad de Valencia, Burjassot, Spain; Instituto de Investigación Sanitaria INCLIVA, Spain

## Abstract

**Background:**

The clinical investigation of human brain tumors often starts with a non-invasive imaging study, providing information about the tumor extent and location, but little insight into the biochemistry of the analyzed tissue. Magnetic Resonance Spectroscopy can complement imaging by supplying a metabolic *fingerprint* of the tissue. This study analyzes single-voxel magnetic resonance spectra, which represent signal information in the frequency domain. Given that a single voxel may contain a heterogeneous mix of tissues, signal source identification is a relevant challenge for the problem of tumor type classification from the spectroscopic signal.

**Methodology/Principal Findings:**

Non-negative matrix factorization techniques have recently shown their potential for the identification of meaningful sources from brain tissue spectroscopy data. In this study, we use a convex variant of these methods that is capable of handling negatively-valued data and generating sources that can be interpreted as tumor class prototypes. A novel approach to convex non-negative matrix factorization is proposed, in which prior knowledge about class information is utilized in model optimization. Class-specific information is integrated into this semi-supervised process by setting the metric of a latent variable space where the matrix factorization is carried out. The reported experimental study comprises 196 cases from different tumor types drawn from two international, multi-center databases. The results indicate that the proposed approach outperforms a purely unsupervised process by achieving near perfect correlation of the extracted sources with the mean spectra of the tumor types. It also improves tissue type classification.

**Conclusions/Significance:**

We show that source extraction by unsupervised matrix factorization benefits from the integration of the available class information, so operating in a semi-supervised learning manner, for discriminative source identification and brain tumor labeling from single-voxel spectroscopy data. We are confident that the proposed methodology has wider applicability for biomedical signal processing.

## Introduction

Brain tumors have a relatively low incidence amongst humans as compared to other more widespread cancer pathologies. The clinical investigation of an abnormal mass in the brain frequently starts with its non-invasive characterization, typically with a Magnetic Resonance Imaging (MRI) study. This is widely used for determining the tumor extent for surgical and radiotherapy planning and for the post-therapy monitoring of tumor recurrence or progression to higher grade. MRI can provide an initial diagnosis of an intracranial mass lesion with variable sensitivity and specificity depending on tumor type [1], [2]. Magnetic Resonance Spectroscopy (MRS) is a complementary MR measurement modality that is increasingly used as a non-invasive method of classifying brain lesions [3]–[5]. Unlike anatomical imaging, spectroscopy provides insight into the biochemistry of tissue through a discrete signal in the frequency domain that reflects the relative abundance of several low molecular weight metabolites, lipids and macromolecules in the millimolar range of concentration.

MRS has already been used in computer-based systems for diagnostic decision support [6]–[9], building on the increasing availability of data in electronic format [10]–[14]. However, for brain tumors and, more specifically, glial tumors, the computer-based discrimination of the grade or the specific subtype of tumor still leaves a “grey zone” of uncertainty between class labels [15]–[17], even after taking into account the spectral resonances characteristic of known metabolites.

The MRS data analyzed in the current work are *single-voxel* (SV) comprising, for each patient, two spectra at slightly different acquisition conditions corresponding to a cubic volume defined by 1.5–2 cm sides, located within the tumor mass. The aim of this study is to separate the constituent source signals (so that they can be separately identified and quantified), guided by the prior labels of tissue class membership assigned to individual spectra, on the assumption that the sources are mixed linearly in each SV spectral measurement. This will provide a quantification of relative class membership that would account for the heterogeneous mix of tissue types within the voxel, thus improving on the more simplistic homogeneous class assignment of the spectrum as a whole. The role of source identification is important because, even within a single voxel, a heterogeneous mix of tissue types may be present. The distribution in the strength of the individual sources is a useful way to resolve ambiguities that arise from tumor heterogeneity.

Previous research has attempted to separate the MRS constituent source signals by applying Independent Component Analysis (ICA) [18], [19] in a fully unsupervised manner, that is without using prior information regarding tumor type and grade. More recently Non-negative Matrix Factorization (NMF) techniques [20], [21] have demonstrated potential benefits from constraining tissue constituents to non-negative mixtures of the source signals, so aiding interpretation [22]–[24]. In [22], a variant called constrained-NMF was used for the analysis of source spectra from MR chemical shift imaging (CSI) data from human brain. In [23], an alternating non-negativity constrained least squares implementation was applied to the analysis of spectra acquired from brain tumors with High Resolution Magic Angle Spinning (HR-MAS). The use of class information in a discriminant version of NMF [25] for brain tumor categorization was also recently studied in [26].

In [24], different variants of NMF, with different initialization conditions, were investigated for the analysis of an international, multi-center database that incorporates MRS data associated with a range of human brain tumor types [10]. This study concluded that the unsupervised analysis of SV MRS data from human brain tumors using a more recent method, Convex-NMF [27], identifies a smaller number of sources that can be confidently recognized as representing brain tumor types or healthy tissue in a way that other source extraction methods, including other NMF variants, cannot detect with the same degree of specificity. Furthermore, the accuracies of the labels inferred for each patient were comparable to traditional supervised classifiers developed for the same datasets.

In a subsequent study [28] we proposed the use of prior knowledge about the analyzed sample to identify sources that best correlate with class prototypes of brain tumors. This is particularly important because the extracted sources are more trusted by clinicians if they are closer to class prototypes. The preliminary results obtained in [28] with synthetic data models of real SV MRS data encouraged us to carry out a more detailed analysis, which has led to this work. In the current study, we have tuned up the methodology to deal even with challenging problems, while retaining interpretability, which is a key requirement in the context of the problem [29]. The prior knowledge used is obtained from the accuracies provided by MRS-based classifiers. We also extend the preliminary work carried out in [28] by using real-world SV MRS data rather than artificially-generated spectra modeled on MRS data and, by testing the methodology against independent test sets, to evaluate the generalizability of the proposed method.

The proposed methodology to guide the separation of the constituent source signals with the use of prior knowledge involves a three-stage approach.

First, a reliable estimation of a probabilistic classifier, from which the probability density function (i.e. probability of class membership) generates a Fisher Information (FI) metric [30]. This is the natural statistical measure of dissimilarity for small perturbations around a given spectrum. This metric enables pairwise distances to be calculated using geodesic paths between distant spectral points, resulting in matrix of spectral dissimilarities obtained in a statistically principled manner. The nature of the FI metric is to amplify distances along important directions, that is to say spectral frequencies that are discriminating between different tissue types, compressing the frequencies that are least informative about class separation [31].

The second step is to map the original data onto a Euclidean projective space so that NMF techniques can be applied. This is done with Multidimensional Scaling methods, by which the spectral points are projected onto a new coordinate space in such a way that the pairwise distances are accurately replicated, so that new data distribution has the same distance structure as the original spectral data, when measured with FI metric. Typical methods are Sammon mapping [32], metric multidimensional scaling [33], or the iterative majorization algorithm [34], [35]. The results are generally insensitive to the particular choice of multidimensional scaling method.

The final step is the application of Convex-NMF for source identification. This implementation is standard but applies to the data in the Euclidean projective space, whose structure captures class discrimination as defined by the probabilistic classifier. Therefore, unlabeled data can be also projected onto the projective space, so positioning themselves in the neighborhood of spectra with similar properties with respect to the probabilistic classification. As this methodology benefits from both supervised and unsupervised modeling stages, we term it semi-supervised.

The remainder of the paper is organized as follows: *Materials and Methods* section, in which the data analyzed are described, along with a brief explanation of the methods that are involved in this study. The basis of the semi-supervised approach for extracting sources is also detailed in this section, as well as the experimental setting. The *Results* section compiles and presents all the experimental results, which are analyzed in detail later on in the *Discussion* section. The most significant findings that emerge from this study are summarized in the *Conclusions* section.

## Materials and Methods

### Ethics Statement

The use of the multicenter data in this study is covered by the original ethical approval obtained by the IRB in each center participating in data collection. In particular, every patient or an authorized relative signed an informed consent form specifically allowing use of his or her data for future scientific research, not just for the original study [10], [36].

### Materials: description of the data

The data analyzed in this study are single-voxel proton MR spectra (SV ^1^H-MRS) acquired *in vivo* from human patients with brain tumors. These data were extracted from INTERPRET, an international multi-center database [10] resulting from the INTERPRET European research project (http://gabrmn.uab.es/interpret) [36]. The data were acquired at 1.5T and at two different echo times, namely short (STE, 20–32 ms) and long (LTE, 135–144 ms), with both modalities available for almost every individual.

This signal acquisition parameter, the time of echo, is used to alter the relative contrast of spectral peaks according to their decay times, so resulting in spectra with different acuity for the detection of specific metabolic peaks. In particular, STE is more sensitive to metabolite signals with short T2 (a MR relaxation time parameter) values, for example, signals from mobile lipids, in addition to which peaks are mostly positive in *in vivo* spectra. On the other hand, LTE spectra are subject to a rotation in the Fourier complex plane which results in some negative peaks, for instance due to the inverted Alanine or Lactate doublets. The experimental benchmarking study reported in the following sections assesses also the differences between the sources extracted in these two different parameter settings, both of which are used in clinical practice.

Class labeling was performed according to the World Health Organization (WHO) system for diagnosing brain tumors by histopathological analysis of a biopsy sample. The modeled data set included measurements at LTE from 20 astrocytomas grade II (A2), 78 glioblastomas (GL) and 31 brain metastases (ME) and at STE from 22 A2, 86 GL and 38 ME. Data were pre-processed as described in [6]. A total of 195 clinically-relevant frequency intensity values measured in parts per million (ppm) were sampled from each spectrum in the [4.24, 0.50] ppm interval and normalized to unit length (UL2) [6].

A further test data set for validation purposes was acquired in three medical centers: Centre Diagnòstic Pedralbes (CDP), Institut d'Alta Tecnologia (IAT) and Institut de Diagnòstic per la Imatge (IDI)-Badalona in Barcelona, Spain. This independent data set was acquired as part of the EU-funded eTUMOUR research project [12]. Pre-processing was the same as for the modeling data. The validation data set comprises STE and LTE spectra from 50 patients and includes 10 A2 and 40 high-grade aggressive tumors (30 GL and 10 ME).

**Figure 1:**
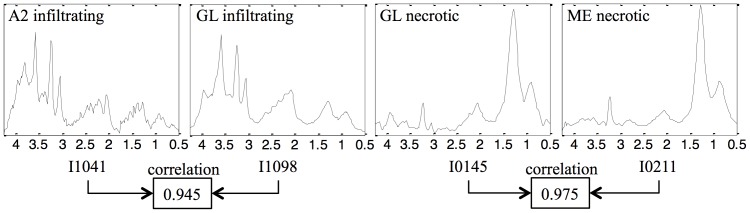
Selected cases from the INTERPRET dataset. Four STE cases selected from the INTERPRET dataset that illustrate the heterogeneity of the GL group, with I0145 showing a necrotic pattern, and I1098 showing an actively proliferating behavior, similar to that of I1041, its low-grade counterpart. These selected cases also illustrate the similarities of I0145 and I0211, which are highly correlated to each other, but are tumor types with different histopathological origins.

The A2 cases are low-grade, grade II on a scale I–IV of the WHO classification [37], corresponding to glial tumors that grow by infiltrating normal brain tissue. They evolve to GL directly or through an intermediate anaplastic glioma stage (WHO grade III), resulting in highly malignant, WHO grade IV tumors. Those who develop through progression of lower grade astrocytomas are called secondary glioblastomas. Primary glioblastomas constitute the vast majority of the glioblastomas, manifesting de novo after a short clinical history, without evidence of a less-malignant precursor tumor [38], [39]. ME are also grade IV tumors, but they are metastases originating from outside of the brain. Grade IV tumors usually have a necrotic pattern, with strong lipid signals that are most evident when obtaining MRS data at short echo times [1]. However, not all GL have this necrotic pattern and some retain a spectral pattern which is overall similar to that of low-grade glial A2 tumors and so might be considered as atypical within their type, or class outliers [40], [41] (see the examples in figure 1).

### Fisher information metric

In this work, the FI measures the change in information about a conditional probability 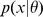 that results from a small perturbation of a particular covariate value 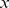 [31], [42]. It is obtained by differentiating the logarithm of the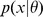 with respect to 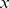 and summing over all possible classifications:
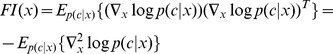
(1)


where 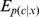 denotes the expectation over the density function 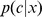 and 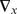 is the gradient with respect to 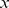.

This definition is the data space equivalent of the more commonly used FI which is about the information carried by the model parameters. In both cases, the FI is derived from a Taylor expansion of the information 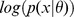
under normality and other constraints discussed in [31]. In the current form, the FI is a function of 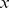 and takes the form of a square matrix of the same dimensionality as 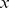, that is, the dimensionality of the data space.

The motivation behind the choice to calculate the FI with respect to the covariates is to directly obtain a dissimilarity measure for comparing spectra using information about their predicted classification. This provides a principled definition of a metric in data space. However this is a local differential metric
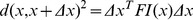
(2)


measuring the distance between two neighboring points 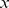 and 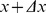. An important property of this metric is that it automatically scales each dimension of the data space according to its degree of relevance with respect to class membership, expanding directions along which 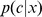 changes rapidly and compressing those where the variation is little. The result is a Riemannian space where the posterior class membership probability changes evenly in all directions.

Our choice of estimator of 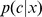
is a Multi-Layer Perceptron (MLP), sometimes called a feed-forward artificial neural network. This is a semi-parametric non-linear probabilistic model of class membership, for which a FI can be derived [31].

### Dataset projection

After estimating the class membership probability, the distance between two points 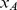 and 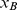 representing different spectra is calculated by minimizing the following path integral 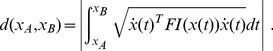
(3)


The path 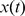 which minimizes this integral defines the geodesic distance between the two locations 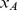 and 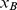. Variable 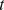, in the interval 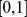, is used to describe the path from 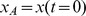 to 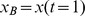; and 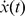 is the derivative of the path with respect to 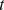. Computational efficient methods to use data sampling to obtain close estimates of geodesic distances are described in [31].

However, this metric space is not flat, in the sense that its metric differs from point to point, therefore many commonly used methods from signal processing cannot be applied unless the data are mapped onto a Euclidean space. To do this while retaining the distance structure generated by the FI matrix requires the application of Multidimensional Scaling methods, which includes the following algorithms:

#### A) Sammon mapping

This algorithm is used to analyze multivariate data by projecting the data points from an original high-dimensional observed space to a space of lower dimensionality [32] such as to preserve the original pairwise distances between observed data points after projection onto the low-dimensional data space. For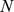 points in a space of dimensionality 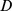 to be projected onto a set of new coordinates in a space of dimensionality 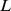, with the distance between points 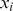 and 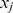 in the original space given by 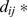
and the distance between their corresponding maps in the 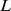-space denoted by 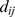, the algorithm minimizes a cost function known as Sammon's stress:
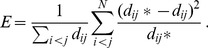
(4)


A random initialization is usually followed by optimization by gradient descent.

#### B) Metric multidimensional scaling

The same fundamental concept of preserving the values of pairwise distances after projection of the original pairwise distances can apply an alternative cost function [43]:
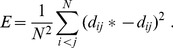
(5)


This is the standard multidimensional scaling algorithm, which is the reason why it is abbreviated here as MDS. This algorithm is applied in this paper since it is the simplest multidimensional scaling method.

#### C) Iterative majorization algorithm

This algorithm, abbreviated in this paper as IMA, expresses the mapping from an original 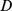-space to a 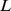-space as a function 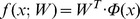, where 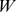
is a 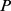-by-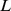 matrix containing the free parameters, and 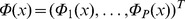 contains the values of 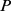 basis functions 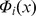. The mapping defined by 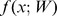 is a linear combination of these basis functions, which can in turn be linear or non-linear. In this work, we have used 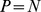 with 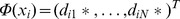, where 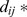 is the Fisher distance between points 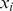 and 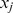. The method tries to minimize the error function
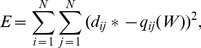
(6)


where 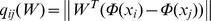, with respect to the weights 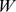
using the iterative majorization algorithm. More detail on this procedure can be found in [44].

### Convex Non-negative Matrix Factorization

In NMF methods, the data matrix 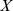
has dimensions 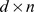, where 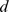 is the number of covariates, in our case the number of selected frequencies to represent each voxel, and 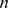 is the number of observations. The aim is to factorize the data matrix into two non-negative matrices, one comprising component sources or basic spectra 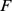, parameterized with dimensions 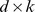, where 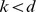 is the number of sources, and the other containing the corresponding scores for each vector 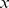, stored in the so-called the mixing matrix 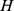 with dimensions 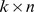, such that the product of these two matrices provides a good approximation to the original data matrix, in the form: 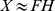. However, in our case LTE spectra include negative components, therefore it is not appropriate to constrain the spectral sources to be non-negative.

Convex-NMF is the algorithmic variant considered in this study, where the source matrix is also factorized into a non-negative mixture of the original data points, 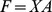
where 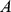 is the unmixing matrix, an auxiliary adaptive weight matrix that fully determines 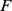, so that 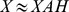. Now the property of non-negativity, which in mathematical terms constrains the optimization process to a convex search space, applies both to the identification of sources from the data and to the representation of the data using scores corresponding to each source.

The constraints of non-negativity are implemented through the use of multiplicative updating algorithms for the key matrices 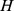 and 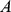 as follows [27]:
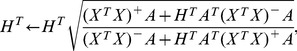
(7)

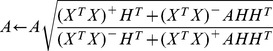
(8)


where 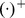 is the positive part of the matrix, where all negative values become zeros; and 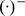 is the negative part of the matrix, where all positive values become zeros.

As proposed in [27], 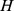 can be fixed to update 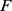 as follows:
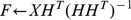
(9)


NMF methods unavoidably converge to local minima. The extracted NMF bases will be slightly different for different initializations. In this study, K-means clustering was applied as proposed in [27], having proved to be the best choice in a previous study [24]. Therefore, 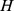
is initialized as 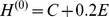, where 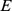 is a matrix with all its elements equal to one, and 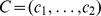 is filled with the cluster indicators, which are based on the cluster indices of each point, such that 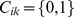 and the ones indicate cluster membership. 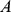 is initialized as 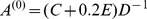, where 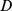 is a diagonal matrix with each element being the number of points in each cluster. The algorithm was considered to have converged when the reconstruction error between successive iterations changed by less than 10^−5^.

In our view Convex-NMF is especially well suited to the analysis of MRS data for the following two reasons:

- The factorization of the source matrix means that Convex-NMF does not require any *ad hoc* distortion of the observed signal in order to enforce non-negativity constraints, in contrast to other implementations of NMF.
- Restricting 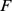 to convex combinations of the columns of 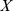 is a unique feature of Convex-NMF that brings about an interesting result: sources can be considered as cluster centroids or, more abstractly, as *representatives* or *prototypes* of the groupings in which the observed data are naturally structured.

As shown in [27], the results of Convex-NMF, if seen as an unsupervised clustering procedure, often agree with those provided by the well-known K-means algorithm [45]. In fact, it is proven in [27] that Convex-NMF is a relaxation of the K-means algorithm. Interestingly, Convex-NMF is bound to generate sparse mixing matrices 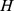 i.e. with many elements taking values close to zero, which is a desirable property for cluster indicators. As a result, the sources obtained by Convex-NMF are likely to be interpretable and similar to data group centroids.

### Semi-supervised approach for extracting source signals

As outlined in the introduction, the purpose of this study is to investigate the potential of using prior knowledge derived from class membership of the spectra to assist the extraction of tissue type-specific MRS signal sources. The methodology proposed involves three main stages and, in a nutshell, can be described as follows:

(i) Definition of a FI metric to model pairwise similarities and dissimilarities between data points, using a MLP classifier to estimate the conditional probabilities of class membership.
(ii) Approximation of the empirical data distribution in a Euclidean projective space in which NMF-based techniques can be applied.
(iii) Application of Convex-NMF for the source decomposition of the data.

### Experimental settings

The experiments of this study involve four approaches: 1) Fully unsupervised extraction of the MRS sources, using Convex-NMF; and 2-4) Semi-supervised extraction of the MRS sources, using, in turn, Sammon mapping, MDS, and IMA, prior to the use of Convex-NMF. With this we aimed to, first, compare the performance of the unsupervised and semi-supervised approaches and, second, compare different alternative semi-supervised approaches. Different problems of brain tumor type classification were considered for experimentation, paying special attention to the quality of the sources obtained and the accuracy of the results.

**Figure 2:**
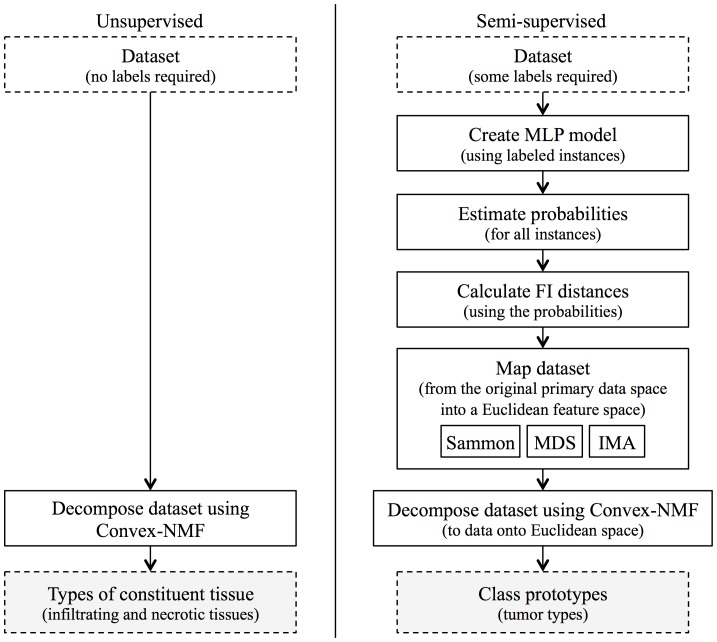
General representation of the analytical approaches investigated in this study. General representation of the unsupervised and semi-supervised approaches analyzed in this study for extracting specific MRS sources in human brain tumors.

For the unsupervised approach (see figure 2, left column), the data matrix was built with the cases of the tumor types involved in each experiment, queried from the INTERPRET dataset. Convex-NMF with K-means initialization was the used for the decomposition of the dataset of spectra into the mixing matrix and the sources. The interpretation of each source was made by reference to the average spectra of the corresponding tumor types, as in [24]. The method was set up to calculate source signals that would represent the constituent tissue types, such as *actively proliferating tissue* (mainly characterized by high levels of choline, and low levels of creatine and mobile lipids) and *necrotic tissue* (high levels of mobile lipids/lactate), as in [24]. This was intended to capture the separation between the constituent tissue types involved. Actively proliferating tissue can be found in A2 and some GL, while necrotic tissue can be found mainly in some GL and ME. After calculating the source signals and the mixing matrix, labels for each case were then generated in a fully unsupervised mode. For this, the values of the mixing matrix were used, after correcting any scaling artifact of the sources and compensating the mixing matrix accordingly. Each class label was subsequently assigned according to the source that had the highest value in the mixing matrix.

For the semi-supervised approaches (see also figure 2, right column), two thirds of the INTERPRET cases (randomly selected) were used to create the MLP model, which was assessed with the remaining third of these cases. This model was used to estimate the conditional probabilities of class membership for each case, which were then used to define the FI metric. From this, three variants of data projection methods were investigated (Sammon mapping, MDS, and IMA), and Convex-NMF with K-means initialization was used for the decomposition of the projected dataset onto the Euclidean space. The interpretation of the sources and the labeling procedure were implemented as for the unsupervised approach. For this set of experiments, source signals were calculated under the hypothesis that they will represent classes or tumor types, not necessarily constituent tissue types. That is, the assistance provided by the prior knowledge to decompose the data is expected to produce sources that resemble class prototypes or tumor types.

An independent test set (the eTUMOUR cases) was used to further validate the generalization capabilities of the obtained sources to label new cases, that is, the capability of correctly labeling unseen, out-of-sample, data cases. Equation (9) provided us with a mechanism to determine the extent to which a fixed set of sources are encoded in a new data set, facilitating the calculation of the corresponding new mixing matrix, and with it, the chance to provide labels for the new cases. Thus, we fixed the sources calculated in the previously described four approaches, and calculated the new mixing matrices for the independent test set.

The experiments involved three tumor types from MRS acquired both at STE and LTE. Firstly, we attempted binary classification for three different brain tumor diagnostic problems, namely A2 *vs*. GL; A2 *vs*. ME; and GL *vs*. ME. The choice of these specific problems at both time of echo acquisition conditions ultimately aimed to find answers to the following questions (figure 1): 1) (A2 *vs*. ME): Are grades (II *vs.* IV) well differentiated? 2) (A2 *vs*. GL): Are grades still well recognized when one of them (GL) is heterogeneous and seemingly overlapping with the other? 3) (GL *vs*. ME): To what extent two high grade tumors (grade IV) can be differentiated? Subsequently, we attempted to discriminate A2 from a superclass containing GL and ME that we call *aggressive* (AG), to evaluate the ability of the methods to differentiate grades (II *vs.* IV) when the grade IV superclass comprises two different tumor types (GL and ME), one of them diverse and visually overlapping with the other grade (A2 *vs*. AG).

Firstly, two source signals were calculated for each classification problem, using the different approaches under study, i.e. fully unsupervised using Convex-NMF, and semi-supervised using the three dataset-projection methods mentioned before (Sammon, MDS, and IMA) prior to Convex-NMF. The fully unsupervised method aimed to extract the constituent tissue types involved in each classification problem, while the semi-supervised ones aimed to extract the *class prototypes* of these classification problems. Given that previous research [24] concluded that two sources were needed to represent the constituent tissue types of heterogeneous classes such as glioblastomas, three source signals were also calculated in the fully unsupervised approach for the discrimination problems A2 *vs*. ME and A2 *vs*. GL. Therefore, in these discrimination problems, one of the sources would represent the A2 class, while the other two would represent, in turn, the GL and ME classes. In the case of the discrimination between GL and ME, it is not clear whether more than two sources would be needed, and what would they represent. Subsequently, three sources were also calculated for the classification problem A2 *vs*. AG in both the fully unsupervised and semi-supervised approaches.

The quality of the sources was determined in terms of how similar they are compared to the mean spectrum of the corresponding class. Similarity was assessed using the correlation between the resulting sources and mean spectra of the classes (tumor types) involved. Calculating the correlation provided us with an indicator of the extent to which each source is tumor-type specific.

The accuracy of the labeling process (for all the methods and diagnostic problems used to assess source extraction) was measured as the ratio of correctly classified cases out of the total number of instances. The balanced error rate (BER) [19] was also calculated. It should provide a more reliable figure of merit in problems with strong class unbalance.

## Results

In this section, we compile and present all the experimental results. The objective of the experiments carried out for this study was twofold: first, the assessment of the ability of the proposed methodology to extract tissue type-specific MRS sources more accurately than previous fully unsupervised approaches and; second, the evaluation of the former as a basis to produce more robust classifiers.

### Source signals

**Figure 3:**
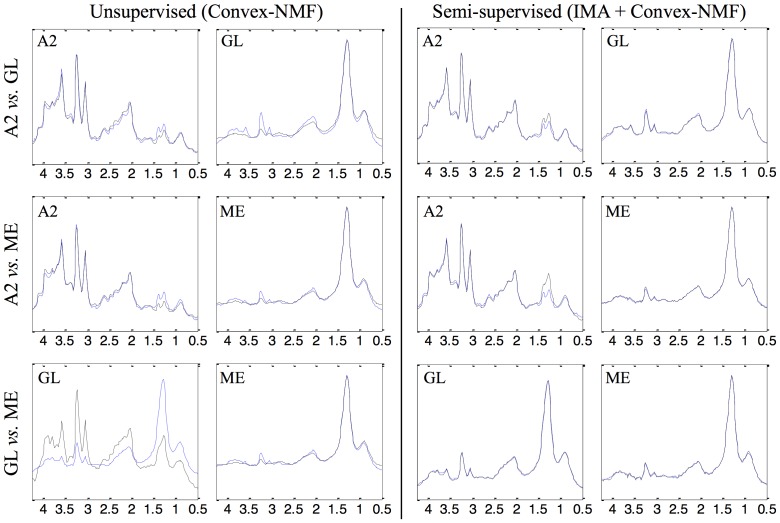
Sources extracted through unsupervised and semi-supervised methods, at STE. Sources extracted for all the classification problems using the training data at STE, for two of the approaches: Convex-NMF (unsupervised), and IMA + Convex-NMF (semi-supervised). The blue spectra indicate the mean of the classes involved. Horizontal axis, for all plots: frequency in ppm scale. Vertical axis, for all plots: UL2 normalized intensity. The range of the vertical scales is fixed for each experiment and is the same for comparative purposes.

**Figure 4:**
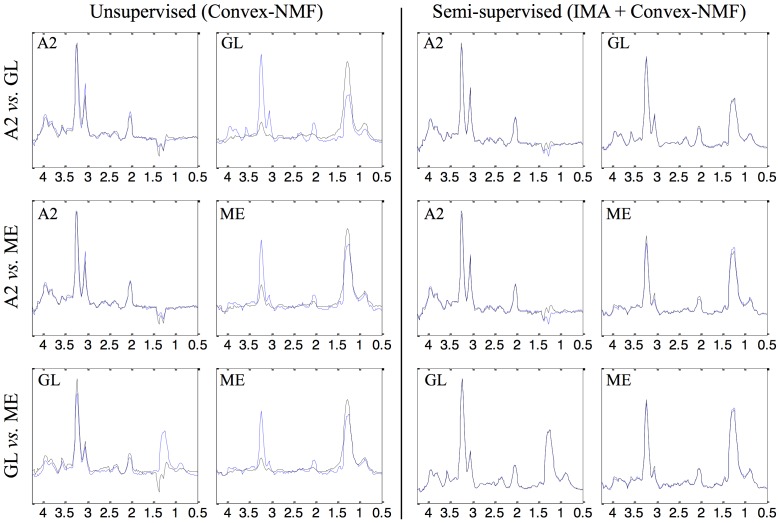
Sources extracted through unsupervised and semi-supervised methods, at LTE. Sources extracted for all the classification problems using the training data at LTE, for two of the approaches: Convex-NMF (unsupervised), and IMA + Convex-NMF (semi-supervised). The blue spectra indicate the mean of the classes involved. Axes labels and representation as in figure 3.

Table 1 compiles the results of the correlations between the extracted sources and the mean spectra of the classes involved for the different approaches. Figures 3 and 4 are graphical illustrative examples of the obtained sources in the experiment with all the classification problems at short and long time of echo (TE), respectively, for two of the approaches: Convex-NMF (unsupervised), and IMA + Convex-NMF (semi-supervised). The blue spectra indicate the mean of the classes involved in each experiment, to be used as reference. IMA + Convex-NMF was the pair selected to present the sources calculated in a semi-supervised way, given that it generally yielded the highest correlations between the mean spectrum of the tumor types and the corresponding extracted sources (see table 1).

**Table 1:** Correlations between the sources and the average spectra.

		STE Convex	STE Sammon Convex	STE MDS Convex	STE IMA Convex	LTE Convex	LTE Sammon Convex	LTE MDS Convex	LTE IMA Convex
A2 *vs.* GL	A2	0.988	0.741	0.935	0.994	0.977	0.947	0.997	0.995
	GL	0.979	0.999	1.000	0.999	0.607	0.999	1.000	0.999
A2 *vs.* ME	A2	0.988	0.931	0.915	0.981	0.990	0.981	0.995	0.986
	ME	0.994	0.999	0.998	1.000	0.872	0.994	0.999	0.993
GL *vs.* ME	GL	0.972	0.999	1.000	0.999	0.776	0.997	0.998	1.000
	ME	0.994	0.994	0.998	0.999	0.831	0.995	0.996	0.998

Table cells should be read as the correlations between the sources and the average spectra (see figures 3 and 4) of the different tumor types. The results of the best performing method for each classification problem are underlined. The latter is measured as the highest average value between the two correlations. When this highest average value is obtained by more than one method, all their corresponding results are underlined.

### Classification results

We next report the results of the unsupervised labeling process: That is, the assignment of class labels (tumor types) to each of the cases using the extracted sources. Bear in mind that in the proposed semi-supervised approaches of this study, class labels are used only to aid the source extraction, but the final labeling process remains unsupervised.

Tables 2 and 3 show the accuracies of the labeling process for all the methods and the diagnostic problems A2 *vs.* GL, A2 *vs.* ME, and GL *vs.* ME, at STE and LTE, respectively, when two source signals were calculated. The extracted sources from the training dataset for the different classification problems (see tables 1–3 and figures 3 and 4), were also used to provide labels for the independent test set, as detailed in the Methods section. The accuracies of the labeling process obtained for this independent test set are shown in tables 4 and 5, for STE and LTE, respectively.

**Table 2:** Labeling accuracy results obtained for the training set at STE.

		Convex-NMF	Sammon + Convex-NMF	MDS + Convex-NMF	IMA + Convex-NMF
A2 *vs.* GL	Total	88.0% (95/108)	97.2% (105/108)	97.2% (105/108)	98.1% (106/108)
	A2	100.0% (22/22)	100.0% (22/22)	100.0% (22/22)	95.5% (21/22)
	GL	84.9% (73/86)	96.5% (83/86)	96.5% (83/86)	98.8% (85/86)
	BER	0.076	0.017	0.017	0.029
A2 *vs.* ME	Total	96.7% (58/60)	96.7% (58/60)	98.3% (59/60)	100.0% (60/60)
	A2	100.0% (22/22)	100.0% (22/22)	100.0% (22/22)	100.0% (22/22)
	ME	94.7% (36/38)	94.7% (36/38)	97.4% (37/38)	100.0% (38/38)
	BER	0.026	0.026	0.013	0.000
GL *vs.* ME	Total	75.8% (94/124)	81.5% (101/124)	83.1% (103/124)	90.3% (112/124)
	GL	70.9% (61/86)	77.9% (67/86)	77.9% (67/86)	94.2% (81/86)
	ME	86.8% (33/38)	89.5% (34/38)	94.7% (36/38)	81.6% (31/38)
	BER	0.211	0.163	0.137	0.121

Summary of the labeling accuracy obtained for the training set, for all the discrimination problems at STE. They include the accuracy (total and by tumor type); the number of correctly labeled samples from the total, in parentheses; and BER of the classification. The highest total accuracy and the lowest BER for each classification problem are underlined.

**Table 3:** Labeling accuracy results obtained for the training set at LTE.

		Convex-NMF	Sammon + Convex-NMF	MDS + Convex-NMF	IMA + Convex-NMF
	Total	55.1% (54/98)	98.0% (96/98)	98.0% (96/98)	98.0% (96/98)
	A2	100.0% (20/20)	95.0% (19/20)	95.0% (19/20)	95.0% (19/20)
	GL	43.6% (34/78)	98.7% (77/78)	98.7% (77/78)	98.7% (77/78)
	BER	0.282	0.031	0.031	0.031
A2 *vs.* ME	Total	80.4% (41/51)	100.0% (51/51)	100.0% (51/51)	100.0% (51/51)
	A2	100.0% (20/20)	100.0% (20/20)	100.0% (20/20)	100.0% (20/20)
	ME	67.7% (21/31)	100.0% (31/31)	100.0% (31/31)	100.0% (31/31)
	BER	0.161	0.000	0.000	0.000
GL *vs.* ME	Total	60.6% (66/109)	76.1% (83/109)	97.2% (106/109)	95.4% (104/109)
	GL	60.3% (47/78)	69.2% (54/78)	100.0% (78/78)	97.4% (76/78)
	ME	61.3% (19/31)	93.5% (29/31)	90.3% (28/31)	90.3% (28/31)
	BER	0.392	0.186	0.048	0.061

Summary of the labeling accuracy obtained for the training set, for all the discrimination problems at LTE. They include the accuracy (total and by tumor type); the number of correctly labeled samples from the total, in parentheses; and BER of the classification. Highest total accuracy and lowest BER underlined as in table 2.

**Table 4:** Labeling accuracy results obtained for the test set at STE.

		Convex-NMF	Sammon + Convex-NMF	MDS + Convex-NMF	IMA + Convex-NMF
	Total	80.0% (32/40)	77.5% (31/40)	80.0% (32/40)	85.0% (34/40)
	A2	100.0% (10/10)	100.0% (10/10)	100.0% (10/10)	100.0% (10/10)
	GL	73.3% (22/30)	70.0% (21/30)	73.3% (22/30)	80.0% (24/30)
	BER	0.133	0.150	0.133	0.100
A2 *vs.* ME	Total	90.0% (18/20)	85.0% (17/20)	85.0% (17/20)	90.0% (18/20)
	A2	100.0% (10/10)	100.0% (10/10)	100.0% (10/10)	100.0% (10/10)
	ME	80.0% (8/10)	70.0% (7/10)	70.0% (7/10)	80.0% (8/10)
	BER	0.100	0.150	0.150	0.100
GL *vs.* ME	Total	62.5% (25/40)	55.0% (22/40)	62.5% (25/40)	70.0% (28/40)
	GL	63.3% (19/30)	56.7% (17/30)	63.3% (19/30)	73.3% (22/30)
	ME	60.0% (6/10)	50.0% (5/10)	60.0% (6/10)	60.0% (6/10)
	BER	0.383	0.467	0.383	0.333

Summary of the labeling accuracy obtained for the test set, for all the discrimination problems at STE. They include the accuracy (total and by tumor type); the number of correctly labeled samples from the total, in parentheses; and BER of the classification. Highest total accuracy and lowest BER underlined as in table 2.

**Table 5:** Labeling accuracy results obtained for the test set at LTE.

		Convex-NMF	Sammon + Convex-NMF	MDS + Convex-NMF	IMA + Convex-NMF
	Total	40.0% (16/40)	65.0% (26/40)	67.5% (27/40)	65.0% (26/40)
	A2	100.0% (10/10)	100.0% (10/10)	100.0% (10/10)	100.0% (10/10)
	GL	20.0% (6/30)	53.3% (16/30)	56.7% (17/30)	53.3% (16/30)
	BER	0.400	0.233	0.217	0.233
A2 *vs.* ME	Total	70.0% (14/20)	75.0% (15/20)	75.0% (15/20)	75.0% (15/20)
	A2	100.0% (10/10)	100.0% (10/10)	100.0% (10/10)	100.0% (10/10)
	ME	40.0% (4/10)	50.0% (5/10)	50.0% (5/10)	50.0% (5/10)
	BER	0.300	0.250	0.250	0.250
GL *vs.* ME	Total	80.0% (32/40)	80.0% (32/40)	82.5% (33/40)	82.5% (33/40)
	GL	86.7% (26/30)	86.7% (26/30)	90.0% (27/30)	90.0% (27/30)
	ME	60.0% (6/10)	60.0% (6/10)	60.0% (6/10)	60.0% (6/10)
	BER	0.267	0.267	0.250	0.250

Summary of the labeling accuracy obtained for the test set, for all the discrimination problems at LTE. They include the accuracy (total and by tumor type); the number of correctly labeled samples from the total, in parentheses; and BER of the classification. Highest total accuracy and lowest BER underlined as in table 2.

Table 6 shows the accuracies of the labeling process obtained when 3 sources were calculated in a fully unsupervised way, for the classification problems A2 *vs.* GL, and A2 *vs.* ME, at STE and LTE, respectively. In these two discrimination problems, one of the sources represents the actively proliferating tissue in A2, while the other two mainly represent the grade IV tumor types. Table 7 shows the results of the labeling process obtained for A2 *vs.* AG (GL+ME), in both unsupervised and semi-supervised ways, when calculating 3 sources, also at STE and LTE, respectively. For these latter results only one of the semi-supervised variants studied was used (IMA + Convex-NMF), for illustration purposes. Also for illustration purposes, table 8 shows how three sources extracted in an unsupervised way for the problem GL *vs.* ME represent these two tumor types, at both STE and LTE.

**Table 6:** Labeling accuracy results obtained with 3 sources, unsupervised (Convex-NMF).

		STE, Training set	STE, Test set	LTE, Training set	LTE, Test set
A2 *vs.* GL	Total	90.7% (98/108)	90.0% (36/40)	79.6% (78/98)	60.0% (24/40)
	A2	95.5% (21/22)	100.0% (10/10)	100% (20/20)	100.0% (10/10)
	GL	89.5% (77/86)	86.7% (26/30)	74.4% (58/78)	46.7% (14/30)
	BER	0.075	0.067	0.128	0.267
A2 *vs.* ME	Total	96.7% (58/60)	85.0% (17/20)	88.2% (45/51)	85.0% (17/20)
	A2	100.0% (22/22)	100.0% (10/10)	100.0% (20/20)	100.0% (10/10)
	ME	94.7% (36/38)	70.0% (7/10)	80.6% (25/31)	70.0% (7/10)
	BER	0.026	0.150	0.097	0.150

Summary of the labeling accuracy obtained for the training and test set when three sources were calculated in a fully unsupervised way (Convex-NMF), for two discrimination problems at STE and LTE. They include the accuracy (total and by tumor type); the number of correctly labeled samples from the total, in parentheses; and BER of the classification.

**Table 7:** Labeling accuracy results obtained with 3 sources, semi-supervised and unsupervised, for A2 *vs.* AG (GL+ME).

		STE, Training set	STE, Test set	LTE, Training set	LTE, Test set
A2 *vs.* AG	Total	89.7% (131/146)	86.0% (43/50)	77.5% (100/129)	60.0% (30/50)
Unsupervised	A2	95.5% (21/22)	100.0% (10/10)	100.0% (20/20)	100.0% (10/10)
	AG	88.7% (110/124)	82.5% (33/40)	73.4% (80/109)	50.0% (20/40)
	BER	0.079	0.088	0.133	0.250
A2 *vs.* AG	Total	97.9% (143/146)	84.0% (42/50)	97.7% (126/129)	66.0% (33/50)
Semi-supervised	A2	100.0% (22/22)	100.0% (10/10)	100.0% (20/20)	100.0% (10/10)
	AG	97.6% (121/124)	80.0% (32/40)	97.2% (106/109)	57.5% (23/40)
	BER	0.012	0.100	0.014	0.213

Summary of the labeling accuracy obtained for the training and test set when three sources were calculated in a fully unsupervised way, and a semi-supervised way (IMA+Convex-NMF), for the discrimination problem A2 *vs.* AG (GL+ME) at STE and LTE. They include the accuracy (total and by tumor type); the number of correctly labeled samples from the total, in parentheses; and BER of the classification.

**Table 8:** Representation of the three sources extracted in unsupervised mode for GL+ME.

	STE, Source 1	STE, Source 2	STE, Source 3	LTE, Source 1	LTE, Source 2	LTE, Source 3
GL	15.1% (13/86)	51.2% (44/86)	33.7% (29/86)	50.0% (39/78)	29.5% (23/78)	20.5% (16/78)
ME	5.3% (2/38)	73.7% (28/38)	21.1% (8/38)	32.35 (10/31)	45.2% (14/31)	22.6% (7/31)

Representation of the three sources to the two tumor types (GL and ME) involved. They include the percentage of cases mainly represented by each source (by tumor type), and the number of cases from the total, in parentheses. Sources were extracted in an unsupervised mode using Convex-NMF for the aggressive tumors group (GL + ME), using the training data at both STE and LTE.

## Discussion

### Source signals

In a previous study [46], the abilities of two variants of ICA [47], [48] (J*ADE* [49] and *FastICA* [50]) were assessed from SV MRS data in the identification of the constituent tissue types of brain tumors. ICA showed no advantages over NMF methods. A subsequent study [24] investigated different variants of NMF and concluded that Convex-NMF, in fully unsupervised mode, is able to produce sources that can be confidently recognized as representing brain tumor tissue types in a way that other source extraction methods, including other NMF variants, cannot.

The results reported in table 1 indicate that, in terms of correlation, all four approaches yield very good sources in general, but semi-supervised variants consistently outperformed the unsupervised one in extracting tissue type-specific (tumor type) sources, yielding better results in all the diagnostic problems studied. The higher correlations provided by IMA + Convex-NMF are very noticeable for all cases, at both times of echo.

Figures 3 and 4 show that the agreement between the sources extracted (in black) in a semi-supervised way, and the mean spectra (in blue) of the two classes (in each experiment and echo time) is almost perfect. The source signals calculated unsupervisedly describe better the constituent tissue types, such as the proliferating and viable behavior of the A2, and the predominantly necrotic pattern of the grade IV tumors; whereas the sources calculated in a semi-supervised mode represent the class average prototypes of the tumor types involved far better. The most interesting case is GL *vs.* ME, both at STE and LTE, where the unsupervised method did not perform as well as the semi-supervised ones. This is because GL and ME have a very similar spectral pattern (their mean spectra have correlations of 0.989 at STE and 0.921 at LTE between each other), which also explains why the classification accuracies were lower for these pairs. Both tumor types are mainly characterized by the necrotic tissue. The semi-supervised alternatives managed to obtain very high source correlations even for the GL *vs.* ME problem, due to the additional information that they bring into Convex-NMF in the form of prior knowledge introduced through the Fisher metric. Thus, the use of class information dramatically improves source-class correlation (from 0.776 to 1 in the case of GL).

With respect to the acquisition conditions, the extracted sources seemed to perform similarly in average at both TE, according to the correlations between the average spectra of the tumor types involved and the sources (table 1). Most of them exhibit values above 0.970. The only discrimination problem for which the result is rather different is GL *vs.* ME at LTE, unsupervised, with correlations below 0.850. Low correlations at LTE might be due to the fact that LTE yields fewer metabolites even if with more clearly resolved peaks.

Regarding the use of different data projection approaches (Sammon, MDS, IMA): in at least one case, the source-class correlation is low for one of the methods that include class information in the source extraction process, namely A2 in the A2 *vs.* GL problem with Sammon's mapping. Given that MDS and IMA (and even the unsupervised model) do not yield such a low correlation, the bad result must be put down to the inability of Sammon's mapping to yield an adequate data projection in this particular case.

### Classification results

The classification results obtained using training data (tables 2 and 3) show that the use of the Fisher metric pre-processing before applying Convex-NMF improves the classification performance with respect to the unsupervised approach, independently of whether Sammon mapping, MDS or IMA was used. The total accuracies were always better for the semi-supervised methods, and only in few cases (A2 *vs.* GL at both TE, and GL *vs*. ME at STE) there were disagreements between the different methods on which class was best classified. That is the reason why the BER was calculated, as it allows us to corroborate that semi-supervised methods yielded the smallest balanced errors. IMA + Convex-NMF was the combination that provided the best accuracies in almost all cases except one, GL *vs.* ME at LTE, in which MDS + Convex-NMF was able to correctly label two more cases than the former. Yet, the result yielded by IMA + Convex-NMF for this problem is very good, accounting for 95.4%, and much better than the unsupervised variant with only a 60.6%. Given that the model has no embedded regularization scheme, there is no guarantee against overfitting. For this reason, results for test are provided in tables 4 and 5 and these should be the ones to take into consideration in order to gauge the generalization capabilities of the proposed approach, not the training ones.

The increase on the accuracy of classification of the test dataset (tables 4 and 5) is smaller than in the training set, but still the fully unsupervised variant does not outperform the semi-supervised ones in any of the discrimination problems. In general terms, the results remain stable for the independent test set, in either the unsupervised or the semi-supervised modalities, since they consistently reflect the same performance as the training set at both TE. The semi-supervised variants managed to classify up to three more cases than the unsupervised one at STE and as much as 11 more cases in a particular discrimination problem at LTE, namely A2 *vs.* GL.

The labeling accuracy results for the training data of the A2 class, as reported in table 2 and 3, are in most cases close or equal to 100%. The only exceptions are the result of a single misclassification from a set of 22 spectra at STE or 20 spectra at LTE.

Given that the A2 test set is smaller (10 spectra both at STE and LTE), we consider that the results reported in tables 4 and 5 just reflect a consistently near perfect classification for this class of tumor and that the similarity between training and test results would increase as the number of available cases increased. Beyond that, the near perfect results for this tumor type reflect that the extracted source is extremely class specific. This class, unlike those of aggressive tumors, is very homogeneous according to its MRS (given that it reflects mostly its proliferating pattern, instead of the characteristic mix of proliferating and necrotic of the aggressive tumors).

Other studies have addressed similar problems in the existing literature, for similar data. We report next some of these results for comparative purposes, although the techniques and the evaluation criteria involved are not always the same and, therefore, not straightforwardly comparable.

In [51], aggressive tumors (GL+ME) were discriminated in a supervised way from A2, as first step of a multiclass classifier for data acquired at LTE, with an accuracy of 84.7% in the training set. In the results of the experiments shown in tables 2 and 3, we have studied the discrimination of A2 from GL and from ME separately, and the accuracies obtained in a semi-supervised way achieved 98% and 100%, respectively, also for the same training set. For the same problem, with data acquired at STE, an accuracy of 90.9% was reported in [52], also in a supervised way and for the training set, to be compared with a 98.1% and 100%, respectively, obtained in our study in a semi-supervised way.

GL *vs.* ME has traditionally been considered as a very difficult differentiation problem by SV ^1^H-MRS data, because of their radiological similarity [19], [53]–[56]. As stated by Opstad *et al.* [56], the radiological appearance of intracranial metastases and high-grade gliomas is often similar and dominated, in both cases, by large peak intensities corresponding to neutral lipids, a byproduct of necrosis [53]. This problem has often been circumvented by considering both pathologies as part of a more general class of high-grade malignant tumors [36], [51], [52]. In the literature, some of the best classification results obtained in a supervised way for this pair of classes were published in [53], where accuracies of 75% at STE and 77.5% at LTE for the independent test set were reported. As the validation dataset of the latter and the current studies is the same with exactly the same processing, the results are comparable. In our study, the semi-supervised methods yielded accuracies of 70% with the test set (table 4) at STE, which means that two less cases were accurately labeled. At LTE, the semi-supervised methods yielded accuracies with the test set of 82.5% (table 5), meaning that, in this case, two more cases were correctly labeled.

Up to this point, only the results corresponding to two extracted sources have been discussed. In [24], it was shown that, to discriminate GL and ME from other tumor types such as A2 in a fully unsupervised way, at least two sources are required. This is because Convex-NMF is not always successful in extracting tumor type-specific sources. Accordingly, a minimum of three sources would be needed, one to represent A2, and two others to represent either GL or ME: one for the necrotic core (high mobile lipids) [1], [56], and the other for the cellular part of the tumor (high total choline, indicating high proliferation rate [57]), according to the signal profile and its metabolic interpretation. This would be valid for both echo times, and both sources are needed to accurately recognize SV patterns of GL or ME [24].

This is the reason why three signal sources were calculated in the discrimination problems A2 *vs.* GL and A2 *vs.* ME, at both TE (table 6), in the unsupervised setting. As expected, most results were improved (training and test sets) with respect to the unsupervised results with two sources shown in tables 2–5, except for the test set results of A2 *vs.* ME at STE.

However, when comparing the unsupervised results obtained with three sources (table 6) with those obtained in a semi-supervised way (tables 2–5), we can see that the semi-supervised approaches, with only two sources, still outperform the unsupervised training results at both TE, but not always the test results. A detailed look at the training results for A2 *vs.* GL obtained with IMA+Convex with two sources (tables 2 and 3) reveals an increased accurate classification of up to 8 (7%) and 18 (18%) cases at STE and LTE, respectively, when compared to the unsupervised results with three sources (table 6, training set columns). Similarly, A2 vs. ME shows an increased accurate classification of up to 2 (3%) and 5 (10%) cases at STE and LTE, respectively. However, and as previously mentioned, the semi-supervised approaches with two sources did not always improve on the unsupervised results with three sources for the test dataset, where IMA+Convex failed to recognize two more cases for A2 *vs.* GL at STE (5%) and A2 *vs.* ME at LTE (10%). The exceptions were A2 *vs.* GL at LTE, in which IMA+Convex (with two sources) recognized two more cases (5%) than the unsupervised model (with three); and A2 *vs.* ME with one more case (5%). It is worth noting that this comparison should be considered with caution, given the different number of sources involved.

**Figure 5:**
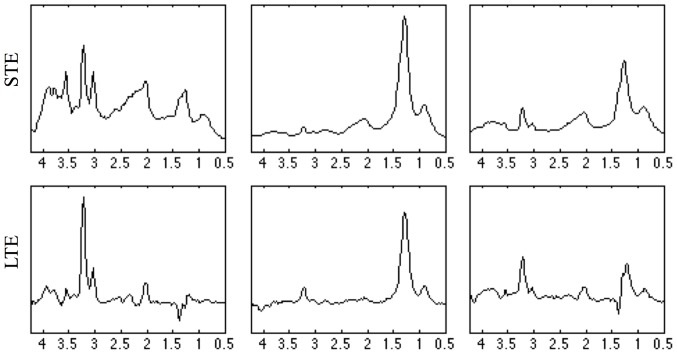
Three sources extracted through unsupervised methods for the AG group. Three sources extracted in unsupervised mode, using Convex-NMF, for the aggressive tumors group (GL + ME), using the training data at both STE and LTE. Axes labels as in figure 3.

In the problem of discrimination between GL and ME, as mentioned in the methods section, it is unclear whether more than two sources would be required, and what they would represent. To illustrate this, three sources were calculated in an unsupervised mode for the aggressive group (GL+ME), as seen in figure 5. The first source (figure 5, column 1) would correspond to non-necrotic GL (see figure 1, case I1098), a minor subtype of the cases at STE (15.1%, see table 8). In this respect, the pattern change shown between STE and LTE sources for the mI/Gly region at ca. 3.55 ppm would suggest high mI content [58], [59] and, accordingly, it would point to secondary glioblastomas as major contributors to this GL subgroup [60]. The mI signal presents J-modulation and its signal decreases the apparent mI peak intensity at ca. 3.55 ppm, whereas the glycine signal, which resonates at the same frequency, does not [58], [59]. Indeed, literature [61]–[63] provides a range of secondary GL percentage (5–8%) close to the percentage given by source 1 (15.1%). Furthermore, second and third sources would represent the ME and the major GL subgroup, containing tissue types from both classes. Source 2 (highly necrotic pattern) represents a higher proportion of ME than source 3 (less necrotic pattern) at both echo times (73.7% ME for source 2 *vs.* 21.1% ME for source 3 at STE; and 45.2% ME for source 2 and 22.6% ME for source 3 at LTE, see table 8). From the previous analysis, we can conclude that even when we can provide an interpretation for the sources extracted, their ability to discriminate one tumor type from the other is reduced due to the degree of mixing of the constituent tissue types. Therefore, semi-supervised approaches play a key role in solving discrimination problems like this one, in which two sources satisfactorily discriminate between the two classes because of their ability to represent class prototypes.

Another classification problem of interest in the literature that involves the tumor types under study is the discrimination between A2 from the superclass AG. When using three sources for this discrimination problem, a semi-supervised approach is able to provide much better results for the training set than the unsupervised approach, with 97.9 *vs.* 89.7% at STE, and 97.7 *vs.* 77.5% at LTE (table 7). The results obtained for the test set show no clear evidence of this advantage, with the unsupervised method being able to classify one more case than the semi-supervised one at STE, and the semi-supervised method being able to represent three more cases than the unsupervised one at LTE. Additionally, the results obtained in a semi-supervised way are better than those presented in [51] and [52], where the reported accuracy was 84.7% at LTE, and 90.9% at STE, to be compared with 97.7% and 97.9%, respectively, obtained in our study.

## Conclusions

The experimental results reported in this study confirm the hypothesis that an unsupervised method ideally suited for source extraction from MRS, namely Convex-NMF, can benefit from the use of the available data class labels to obtain tumor type-specific sources that result in accurate classifiers without any loss in the interpretability of the results.

A novel mechanism to perform non-negative matrix factorization in a semi-supervised manner is provided, by first finding a natural metric to describe the class assignments, and then mapping the data using standard projective methods into an approximate distribution in a Euclidean space where standard projective methods of the source extraction can be applied.

For the data analyzed in this work, the proposed semi-supervised approach yielded the better classification accuracies, both in the training and test datasets, if two sources were employed. Moreover, when interpreted as class prototypes, the extracted sources were of higher quality than those calculated using the unsupervised method. The results were more similar between unsupervised and semi-supervised source extraction-based classification when three sources were employed. However, the semi-supervised approaches were key in problems where the unsupervised extraction of three sources is not being helpful, such as the discrimination of GL from ME. For this problem, the accuracy results obtained using the semi-supervised approach were comparable to the best reported in the literature, with the added value of the interpretability provided by the sources.

In conclusion, the improvements in classification accuracy and accuracy of sources identification, especially in complex tumor type classification problems, are the main advantages of using the additional pre-processing steps when the focus is that of finding tumor-type specific MRS signal sources.

The differences between unsupervised and semi-supervised methods are less apparent when three sources are identified. Theoretical approaches to defining the optimal number of sources should be the subject of further work.
